# Increase in circulating Th17 cells during anti-TNF therapy is associated with ultrasonographic improvement of synovitis in rheumatoid arthritis

**DOI:** 10.1186/s13075-016-1197-5

**Published:** 2016-12-23

**Authors:** Dobrina N. Hull, Helen Cooksley, Shilpa Chokshi, Richard O. Williams, Sonya Abraham, Peter C. Taylor

**Affiliations:** 1 Department of Medicine, Imperial College London, London, UK; 2 Institute of Hepatology, The Foundation for Liver Research, 111 Coldharbour Lane, London, SE5 9NT UK; 3 Kennedy Institute of Rheumatology, Nuffield Department of Orthopaedics, Rheumatology and Musculoskeletal Sciences, University of Oxford, Botnar Research Centre, Windmill Road, Headington, Oxford, OX3 7LD UK

**Keywords:** Ankylosing spondylitis, Anti-TNF, Psoriatic arthritis, Rheumatoid arthritis, T cells

## Abstract

**Background:**

Anti-TNF agents have revolutionised rheumatoid arthritis (RA) treatment; however, a third of patients fail to achieve therapeutic responses. Unexpectedly, studies in murine and human arthritis have indicated that anti-TNF treatment can increase circulating T helper 17 (Th17) cells, but the relationship to treatment response is unclear. To identify immune correlates of anti-TNF treatment response, we conducted a longitudinal study using clinical, ultrasound and T cell assessments.

**Methods:**

Patients with RA (*n* = 25) were studied at protocol visits during the initial 12 weeks of anti-TNF treatment. Improvement in the disease activity score of 28 joints (DAS28) >1.2 defined treatment responders (*n* = 16) and non-responders (*n* = 9). Changes in synovial thickening and vascularity of 10 metacarpophalangeal joints were quantitatively assessed by grey scale and power Doppler ultrasound. The frequency of circulating Th17 cells was determined by IL17 enzyme-linked immunospot assay (Elispot) and flow cytometry (fluorescence-activated cell sorting (FACS)).

**Results:**

The frequency of circulating IL17-producing cells increased significantly 12 weeks after anti-TNF initiation (Elispot median (range) specific spot forming cells (spSFC)/10^6^ 360 (280–645) vs 632 (367 − 1167), *p* = 0.003). The increase in CD4 + IL17+ cells at 12 weeks was confirmed by FACS (median (range) %, 0.7 (0.5–0.9) vs 1.05 (0.6–1.3); *p* = 0.01). The increase in circulating Th17 cells inversely correlated with reduction in synovial vascularity (*r* = -0.68, *p* = 0.007) and thickening (*r* = -0.39; *p* = 0.04). Higher frequencies of circulating Th17 cells at baseline were associated with poorer anti-TNF treatment response defined by ultrasonographic measures.

**Conclusions:**

These results demonstrate a link between changes in circulating Th17 cells with resolution of ultrasonographic features of synovial inflammation and vascularity during anti-TNF treatment. The findings may reflect redistribution of Th17 cells from inflamed joints or TNF-driven regulation of Th17 cell production.

**Trial Registration:**

ClinicalTrials.gov: NCT01060098. Registered 29 January 2010.

**Electronic supplementary material:**

The online version of this article (doi:10.1186/s13075-016-1197-5) contains supplementary material, which is available to authorized users.

## Background

Th17 cells are a highly pro-inflammatory T helper (Th) cell subset, which have been shown to contribute to arthritis pathogenesis [1, 2]. Their signature cytokine, interleukin 17 (IL-17), has pleiotropic effects on effector cells of the immune system and can induce production of other pro-inflammatory cytokines [1], contribute to cartilage damage by promoting release of matrix metalloproteinases, increase osteoclast differentiation leading to bony erosions and mediate angiogenesis in inflamed joints [2–5]. Increased frequencies of Th17 cells and IL17 levels have been found in the peripheral blood of patients with RA compared to healthy controls or patients with osteoarthritis. In addition, Th17 cells are further enriched in RA synovial fluid and tissue, where their levels correlate with inflammatory markers and active synovitis [6–9]. Histological studies have also shown the presence of IL17 in T-cell-rich areas of synovium [3, 6]. Furthermore, synovial tissue IL17 mRNA has been shown to be associated with increased progression of joint damage in RA in a 2-year prospective study [10].

Anti-TNF treatment has revolutionised the management of RA, leading to improvement in signs and symptoms, and slowing progression identified on radiography [11]. However, despite anti-TNF treatment being successful in the majority of patients, 20–30% of patients do not respond or experience significant side effects [12]. Assessing RA disease activity is critically important to be able to adjust treatment regimens with the aim being to ‘treat to target’ and achieve low disease activity or induce remission, and in the long term prevent joint damage [13]. Power Doppler ultrasound (PDUS) has become an invaluable tool for this as multiple studies have demonstrated its increased sensitivity in the detection of synovial inflammation compared with clinical examination alone [14–17]. Moreover, PDUS has been shown to be more sensitive than clinical outcome measures in evaluating treatment response [18–22]. Specifically, PDUS scores have been shown to reflect changes in disease activity during anti-TNF treatment in patients with RA, and reduced scores following therapy are associated with reduced progression of joint damage [19, 22]. However, no data are available to date to investigate the impact of anti-TNF therapy on T cell subsets in RA in relation to changes in synovial thickening and vascularity in the joints.

In collagen-induced arthritis (CIA), a mouse model of RA, amelioration of arthritis during anti-TNF treatment has intriguingly been shown to increase the numbers of Th17 cells in draining lymph nodes, while reducing the numbers of these cells in the inflamed paws [23]. Subsequently two small studies in patients with RA also suggest that anti-TNF treatment may increase circulating Th17 cells, but these studies did not directly investigate the relationship of these changes to treatment response [24, 25]. We have previously reported an increase in peripheral blood Th17 cell numbers by fluorescence-activated cell sorting (FACS) and an increase in IL17-producing cells by enzyme-linked immunospot assay (Elispot) after anti-TNF treatment in patients with ankylosing spondylitis, psoriatic arthritis and RA [26]. In addition, we showed that the kinetics of change in peripheral blood Th17 cells are the same whether or not TNF inhibition is mediated by adalimumab (a monoclonal antibody) or etanercept (a TNFR-2 fusion protein) [26].

In the present study, we extend these observations to longitudinally investigate the relationship between the frequency of circulating Th17 cells during anti-TNF therapy in patients with RA with clinical and morphological changes, in particular synovial thickening and vascularity as assessed by ultrasonography in response to treatment.

## Methods

### Study population

Patients with RA (*n* = 25) with an established diagnosis according to the 1987 American College of Rheumatology criteria [27] were recruited from Imperial College Healthcare National Health Service (NHS) Trust clinics (Table 1). Patients were evaluated at four pre-determined protocol visits - prior to treatment initiation and at 1, 4 and 12 weeks after initiation of treatment with anti-TNF agents. Inclusion criteria included active disease at baseline as defined by the disease activity score of 28 joints (DAS28) >5.1 on two occasions at least 1 month apart, and failed therapy with at least two disease-modifying anti-rheumatic drugs (DMARDs), including methotrexate. Patients were treated with either etanercept 50 mg weekly or adalimumab 40 mg fortnightly as determined by their treating physician. Patients were excluded if they had previously been treated with biologic agents, had inter-current active infection, had undergone a change in the dose of DMARDs in the 4 weeks preceding study entry or had received oral, intramuscular or intra-articular steroids in the preceding 4 weeks.

**Table 1:** Changes in clinical measures of disease activity over 12 weeks on anti-TNF treatment in 25 patients with rheumatoid arthritis

Parameter	Baseline	Week 1		Week 4		Week 12	
	Mean ± SD	Mean ± SD	*P* value	Mean ± SD	*P* value	Mean ± SD	*P* value
DAS28-ESR	5.72 ± 0.84	4.47 ± 1.14	****	4.26 ± 1.13	****	2.87 ± 1.11	****
DAS28-CRP	5.28 ± 0.98	3.98 ± 1.09	****	3.85 ± 1.13	****	2.36 ± 1.04	****
SJC28	7.68 ± 5.36	4.42 ± 3.89	**	4.54 ± 4.28	**	3.00 ± 3.83	***
TJC28	14.88 ± 8.51	8.95 ± 6.66	***	8.76 ± 6.69	**	4.68 ± 4.93	***
CRP	15.74 ± 18.26	5.29 ± 6.39	***	10.75 ± 24.15	***	8.73 ± 16.48	***
ESR	27.21 ± 22.67	20.50 ± 19.36	**	19.96 ± 14.88	**	20.45 ± 16.73	ns

Data are presented as mean ± SD. *DAS28* disease activity score in 28 joints, *ESR* erythrocyte sedimentation rate, *CRP* C-reactive protein, *SJC28* swollen joint count out of 28 joints, *TJC28* tender joint count out of 28 joints

Each time point on treatment was compared to baseline using Wilcoxon matched pairs test; **P < 0.001, ***P < 0.0005, ****P < 0.0001

Patients were defined as responders (*n* = 16) or non-responders (*n* = 9) to anti-TNF based on whether they achieved improvement in the DAS28 score >1.2 from baseline to 12 weeks on treatment, based on European League Against Rheumatism (EULAR) response criteria [28]. Age, disease duration, medications, presence of rheumatoid factor or anti-cyclic citrullinated peptide (Anti-CCP) antibody positivity were obtained from review of medical notes. Disease activity was assessed by the DAS28 score on the day of sample collection and erythrocyte sedimentation rate (ESR) and C-reactive protein (CRP) were determined in the clinical laboratory. Patients also underwent high frequency grey scale and power Doppler ultrasound of 10 metacarpophalangeal (MCP) joints to assess synovial thickening and synovial vascularity at each visit. Subject data are summarised in Table 1.

### Peripheral blood mononuclear cell (PBMC) isolation

Peripheral venous blood was collected in glass tubes containing sodium heparin and PBMC were isolated by density gradient centrifugation using Lympholyte (Cedarlane, Ontario, Canada). PBMC were cryopreserved at a density of 5 − 10/10^6^/ml in heat-inactivated fetal bovine serum (FBS) (Gibco, Paisley, UK) containing 10% dimethyl sulfoxide (Sigma, Gillingham, UK), placed overnight at -80 °C in a cryogenic vessel containing isopentane and subsequently transferred to liquid nitrogen. To minimise intra-assay variability, all measurements at all time points were analysed simultaneously for each patient.

### Elispot assay to quantitate IL17-producing cells

PBMC from the baseline visit and weeks 4 and 12 on treatment were thawed, washed and resuspended at 2 × 10^6^/ml in RPMI containing 10% human AB serum (Sigma). Cell viability by trypan blue was consistently >95%: 2 × 10^5^ cells were cultured in triplicate in RPMI/10%AB serum containing 1 μg/ml anti-CD3 (OKT3 clone, eBiosciences, Hatfield, UK) for 20 hours. PHA (1 μg/ml) or medium alone were used as positive and negative controls respectively. Sterile multiscreen 96-well plates (Millipore, Bedford, MA, USA) were coated with IL17 capture antibody (R&D Systems, Abingdon, UK), used according to manufacturer’s instructions, and incubated at 4 °C for 16 hours. The stimulated cells were transferred to the coated plates for a further 24 hours. The plates were washed and biotinylated anti-IL17 antibody (R&D Systems), used according to manufacturer’s instructions, was added for 2 hours. Streptavidin-AP (R&D Systems), used according to manufacturer’s instructions, was added to the plates for 2 hours at room temperature in the dark. BCIP/NBT solution (R&D Systems) was added for 30 minutes to allow visualisation of coloured spots. The spots were counted using an automated Elispot reader (AID, Strassberg, Germany). The number of specific spot forming cells (spSFC) was determined as the mean number of spots in the presence of stimulation agent minus mean number of spots in wells containing medium only.

### Flow cytometry quantitation of CD4 + IL17-producing cells

Thawed PBMC at a concentration 15 × 10^6^/ml were placed in culture medium (RPMI 1640 supplemented with 10% FBS, 1% penicillin/streptomycin and 1% L-glutamine, all Sigma) and stimulated for 5 hours with phorbol myristate acetate (PMA, 50 ng/ml) and Ionomycin (500 ng/ml) (both Calbiochem, Nottingham, UK) in the presence of 10 μg/ml Brefeldin A (Sigma). Cells were incubated with Aqua-Live/dead fixable dead cell kit (Invitrogen, Paisley, UK), used according to manufacturer’s instructions, for 30 minutes at 4 °C and subsequently stained with anti-CD4-FITC and anti-CD8-PerCP/Cy5.5 (BD Biosciences, Oxford, UK) for 30 minutes at 4 °C before fixation with Cytofix (BD Biosciences). Cells were permeabilised with phosphate-buffered saline containing 1% bovine serum albumin and 0.05% saponin (Sigma) and stained with anti-IL17-PE (BD Biosciences) for 30 minutes. Cells were acquired and analysed on the FACS CantoII using FACS DIVA software (BD Biosciences). Live cells were identified by FSC-A/FSC-W profile and negative staining for Aqua-live/dead kit. Lymphocytes were identified by back-gating from CD8 and using the forward and side scatter profile.

### PDUS image acquisition

At each study visit, patients underwent high-frequency grey scale and power Doppler ultrasound over the dorsum of 10 MCP joints in the transverse plane using the GE Logiq9 machine with a two-dimensional M12L transducer (GE Healthcare, Buckinghamshire, UK) performed by the same sonographer (DH) to ensure consistency. Settings on the machine were kept constant: grey scale frequency 14 MHz, power Doppler frequency 7.5 MHz, Gain 50, PRF 1.4 KHz, wall filter 127 Hz. To standardise image acquisition, the hands were maintained in a position of rest using a splint. The timing of scanning at each visit was within 1 hour of the baseline visit. Images were anonymised and stored for future analysis.

### PDUS image analysis

Anonymised clips and images were analysed by the same assessor (DH) for synovial thickening and vascularity in the transverse plane, by a quantitative scoring method previously described by us using a computerised image analysis system (ImageJ, v1.42q, NIH, Bethesda, USA) [19, 29]. Synovium was defined as an anechoic or hypoechoic region over the dorsum of the joint, visible in the transverse and longitudinal planes. The synovial area measures were made from the first technically qualified image on high frequency grey scale ultrasound. Each PDUS scan consisted of a 5-second movie clip and PDUS measures were made on the image frame at the peak of the PDUS signal. Synovial thickness area (STA) is a count of the number of pixels with synovial hypertrophy within a defined region of interest. The power Doppler area (PDA) is a count of the number of pixels with PDUS signal within the defined region of interest. Extra-articular digital vessels and reflection artefacts were excluded, if present. The individual STA and PDA scores of each MCP were summated to create a total synovial thickening (Trans STA) and synovial vascularity score (Trans PDA), respectively for all 10 MCPs.

### Statistical analysis

The analysed parameters obtained at protocol time points on treatment were compared to baseline using the Wilcoxon signed rank matched-pairs test. Correlation was analysed using Spearman rank. Data were analysed using Prism version 5 (Graphpad Software Inc, La Jolla, USA); *p* values less than 0.05 were considered significant.

## Results

### Patient characteristics and response to therapy

Patients with RA (*n* = 25, 18 female and 7 male; mean age ± SD 57.4 ± 11.7 years; mean disease duration ± SD 10.6 ± 9.2 years) with active disease (mean DAS28 ± SD 5.72 ± 0.84) were followed longitudinally during the initial 12 weeks of treatment with anti-TNF (18 patients were treated with etanercept and 7 patients with adalimumab). Anti-TNF treatment led to a significant reduction in DAS28, as early as 1 week after anti-TNF initiation in the whole cohort, with further reduction at 4 and 12 weeks on treatment (Table 1). There was also a significant improvement in laboratory measures of disease activity, ESR and CRP. Based on EULAR response criteria, 16 patients were classified as good responders and 9 patients as non-responders to anti-TNF therapy at 12 weeks.

### Improvement in synovial thickening and synovial vascularity with anti-TNF therapy

To assess variability in grey scale and PDUS image acquisition, five patients were scanned twice at the same sitting and the intra-class correlation coefficient (ICC) for Trans STA (ICC = 0.99) and Trans PDA (ICC = 0.99) indicated excellent agreement between the two scans confirming the imaging technique was consistent and reproducible. To assess within-scan intra-reader reproducibility in image analysis, 10 anonymised scans were randomly selected and the grey scale and PDUS were re-read in a blinded fashion and the intra-class correlation coefficient for Trans STA (ICC = 0.91) and Trans PDA (ICC = 0.99) indicated excellent agreement.

At baseline, quantitative measures of synovial vascularity correlated positively with disease activity (DAS28, *r* = 0.59, *p* = 0.01). The kinetics of change of both quantitative measures of synovial thickening and synovial vascularity did not differ significantly between those patients treated with etanercept and those with adalimumab and both measures showed improvement with anti-TNF treatment, irrespective of type (data not shown).

**Fig. 1:**
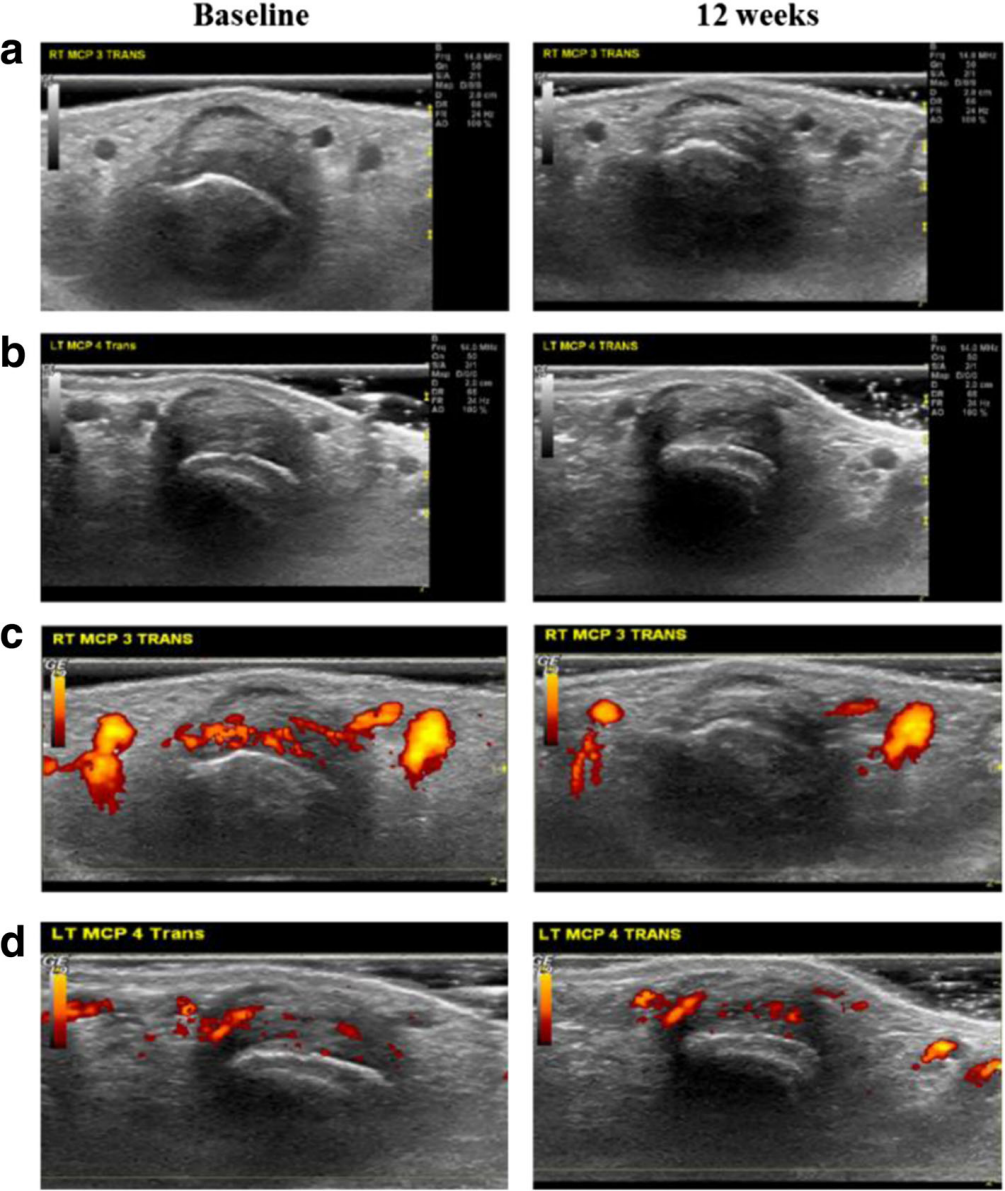
Differences between anti-TNF responders and non-responders in improvement in synovial thickening and vascularity. Representative ultrasound images of changes in synovial thickening (**a** and **b**) and synovial vascularity (**c** and **d**) in a metacarpophalangeal joint of a good responder (**a** and **c**) and poor responder (**b** and **d**) to anti-TNF therapy

The quantitative measures of synovial thickening and synovial vascularity were both able to discriminate between EULAR good responders and non-responders to anti-TNF treatment (Fig. 1). Overall, in the subgroup of anti-TNF responders there was a significant improvement in Trans STA after 4 and 12 weeks of therapy and a significant improvement in Trans PDA after 1, 4 and 12 weeks of treatment, whereas neither measure changed significantly in non-responders during anti-TNF treatment (Table 2). Representative high-frequency grey scale and PDUS images from a EULAR good responder and non-responder patient at baseline and after 12 weeks on treatment are shown in in Fig. 1. Importantly, although in EULAR good responders both Trans STA and Trans PDA measures improved significantly during treatment, these ultrasound measures exhibited different kinetics of change, with earlier and more marked improvement in synovial vascularity during anti-TNF treatment compared to synovial thickening (Table 2).

**Table 2:** Changes in ultrasound measures of synovial thickening and synovial vascularity in treatment responders and non-responders during 12 weeks of anti-TNF treatment

Parameter	Baseline	Week 1		Week 4		Week 12	
	Median (range)	Median (range)	*P* value	Median (range)	*P* value	Median (range)	*P* value
Total synovial thickness area score (10 MCP trans STA)							
*Responders (n = 16)*	203,039 (134,989–311,455)	186,048 (143,073–281,634)	ns	181,288 (115,760–257,808)	*	139,824 (94,708–226,389)	**
*Non-responders (n = 9)*	110,231 (98,185–156,452)	125,946 (114,966–180,770)	ns	117,202 (94,965–220,584)	ns	116,119 (67,559–189,271)	ns
Total synovial power Doppler area score (10 MCP trans PDA)							
*Responders (n = 16)*	3213 (115–6772)	949 (156–2967)	*	114 (17–2310)	**	76 (4.5–1075)	*
*Non-responders (n = 9)*	25 (0–1079)	48 (0–98)	ns	57 (0–169)	ns	87 (12.5 − 780)	ns

Values are presented as median (interquartile range). Comparison of each time point on treatment versus baseline made using Wilcoxon matched pairs test; *p < 0.05, **p < 0.005, *ns* non-significant

### Anti-TNF treatment increases frequency of circulating Th17 cells

**Fig. 2:**
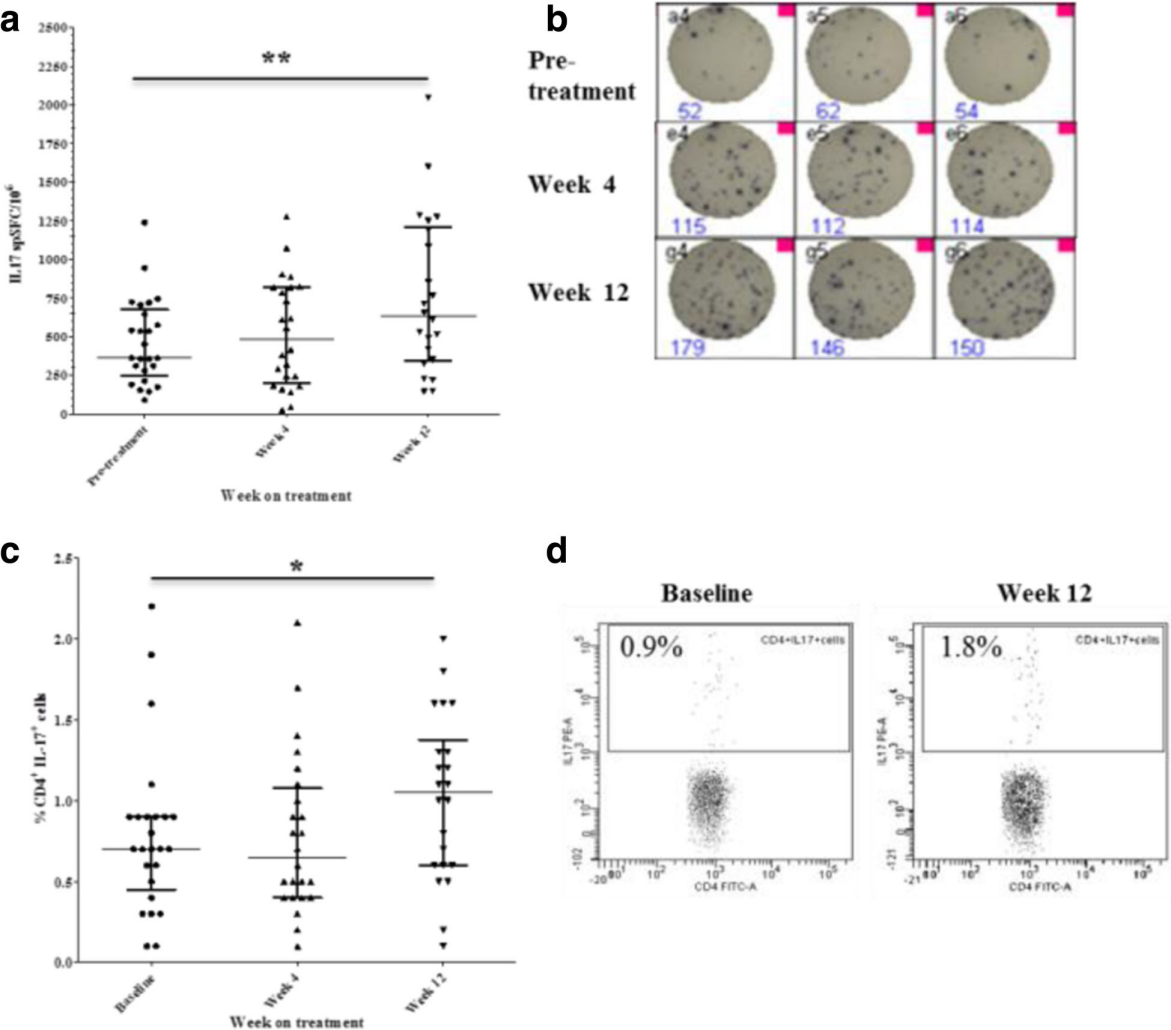
Anti-TNF treatment increases frequency of circulating T helper (Th)17 cells. Change in numbers of IL17-producing peripheral blood mononuclear cells during anti-TNF treatment determined by IL17 enzyme-linked immunospot (Elispot) assay (**a**) and representative IL17 Elispot experimental wells (**b**). PBMC (n = 200,000) were seeded in triplicate in each experiment and stimulated with 1 mg/ml anti-CD3 antibody for 20 hours and the numbers of cytokine-producing cells were enumerated. Change in the frequency of circulating CD4 + IL17+ cells in the peripheral blood of patients with rheumatoid arthritis (RA) determined by flow cytometry (**c**) and representative dot plots (**d**). *Bars* represent median and interquartile range; *p < 0.05, **p < 0.01 versus baseline visit (Wilcoxon matched pairs test). *spSFC/10* ^*6*^, specific spot-forming cells per million PBMC

The frequency of circulating IL17-producing cells (spSFC/10^6^ PBMC by IL17 Elispot assay) increased significantly 12 weeks after anti-TNF initiation (median (range) spSFC/10^6^ 360 (280–645) vs 632 (367–1167), *p* = 0.003) compared to baseline (Fig. 2a, b). To determine the relative proportion of CD4+ cells amongst IL17-producing cells in the Elispot assay, PBMC depleted of CD4+ cells using magnetic beads were tested in three patients with RA. Whole PBMC and a sample of PBMC from the same patients after CD4+ cell depletion were set up in the IL17 Elispot assay. The effectiveness of the depletion and purity of the depleted fraction was verified by flow cytometry and confirmed that the remaining CD4+ cell population was effectively depleted of CD4+ cells (Fig. 1a, Additional file 1: Figure S1). The depletion of CD4+ cells from the PBMC population significantly reduced IL17 responses, demonstrating the great majority of IL17 was produced by CD4+ cells (Fig. 1b and c, Additional file 1: Figure S1).

Using flow cytometry we evaluated the presence of CD4+ cells co-expressing IL17 and IFNγ but consistently found the frequency of this population to be negligible both at baseline and during anti-TNF treatment.

Flow cytometry analysis also confirmed a significant increase in the percentage of circulating CD4 + IL17+ cells 12 weeks after anti-TNF initiation compared to baseline (median (range) %, 0.7 (0.5–0.9) vs 1.05 (0.6–1.3), *p* = 0.01) (Fig. 2c and d).

### Increase in circulating Th17 cells correlates with reduction in joint inflammation

**Fig. 3:**
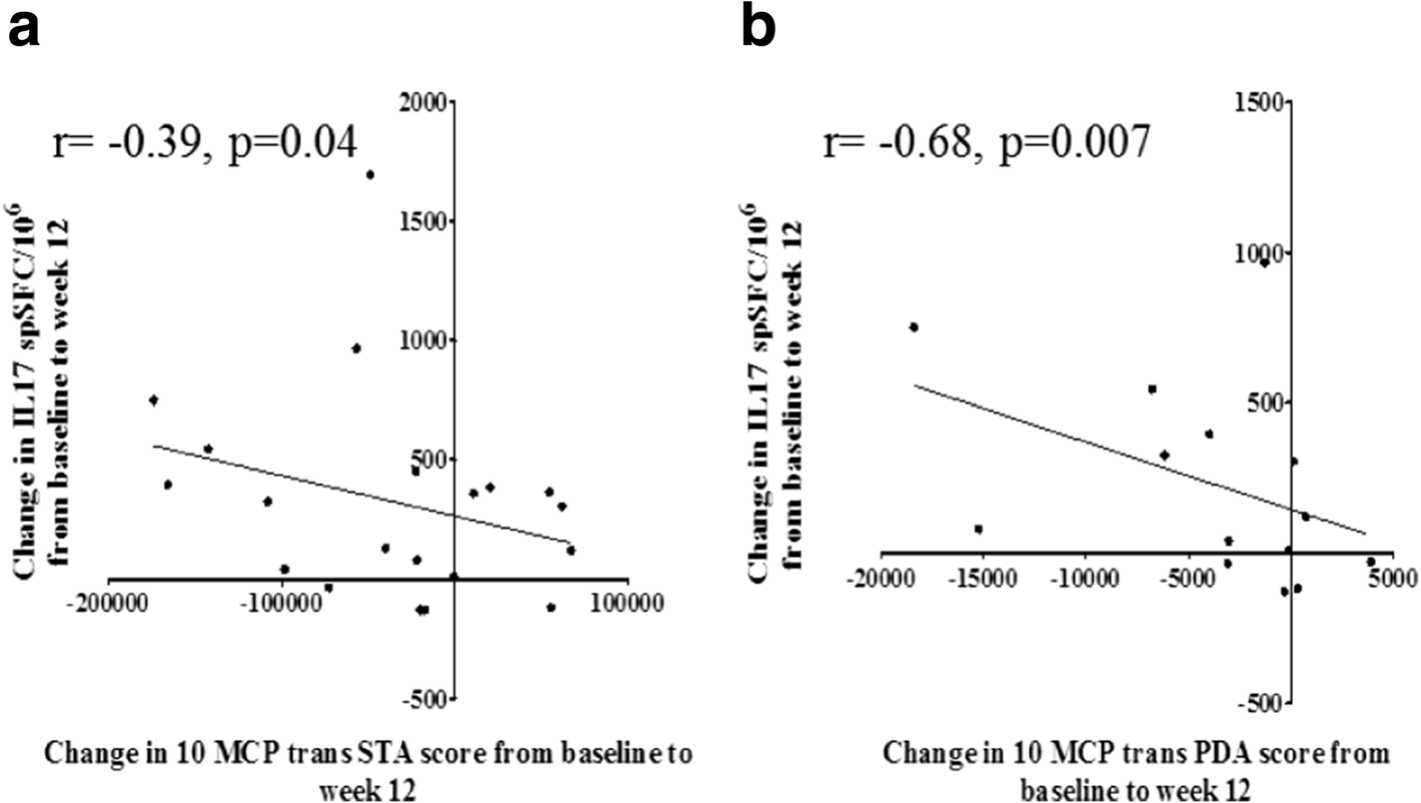
Increase in circulating T helper (Th)17 cells during anti-TNF treatment correlates with improvement in joint inflammation. Negative correlation is shown between the change in frequency of IL17-positive cells (determined by IL17 enzyme-linked immunospot (Elispot) assay) from baseline to week 12 on treatment and the change in quantitative ultrasound scores for synovial thickening (Trans STA) and vascularity (Trans PDA) from baseline to week 12 in patients with rheumatoid arthritis (RA). Correlation was tested using the Spearman’s rank method. *10 MCP Trans PDA* composite transverse power Doppler area score for synovial vascularity of ten metacarpophalangeal joints, *10 MCP Trans STA* composite transverse synovial thickness area score of ten metacarpophalangeal joints. **a** Synovial thickening. **b** Synovial vascularity

Having shown that anti-TNF treatment induces an increase in circulating Th17 cells in patients with RA, we then investigated the relationship between this increase in circulating Th17 cells and clinical and ultrasonographic measures of disease activity. We found significant negative correlation between the change in numbers of IL17-producing cells (by IL17 Elispot) from baseline to week 12 on treatment and the change in Trans STA score (*r* = -0.39, *p* = 0.04) and the change in Trans PDA score (*r* = -0.68, *p* = 0.007) from baseline to 12 weeks on treatment (Fig. 3). These negative correlations suggest that the increase in frequency of circulating Th17 cells occurring during anti-TNF treatment is associated with reduction in synovial thickening and synovial vascularity in patients with RA.

### Higher frequency of circulating Th17 cells at baseline is associated with poor anti-TNF response

**Fig. 4:**
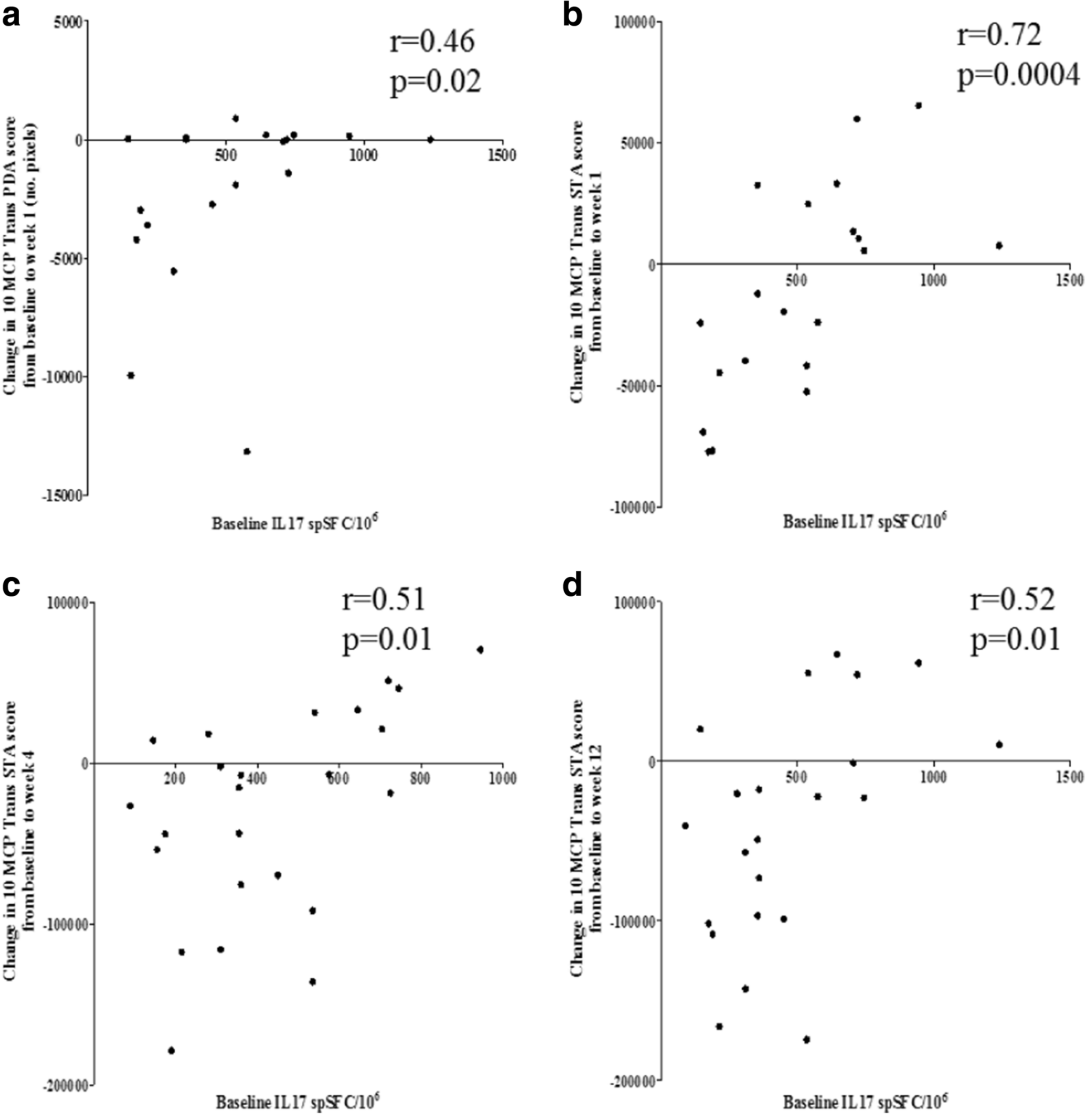
Higher frequency of T helper (Th17) cells at baseline associated with poorer response to anti-TNF treatment. Positive correlation between the frequency of circulating IL17-producing cells (determined by IL17 enzyme-linked immunospot (Elispot) assay) at baseline and the change in quantitative score for synovial vascularity (composite transverse synovial thickness area score of ten metacarpophalangeal joints (*10 MCP Trans PDA score*) from baseline to week 1 on treatment (**a**) in patients with rheumatoid arthritis (RA) with presence of power Doppler signal at baseline (*n* = 19). Positive correlation is shown between the frequency of circulating IL17-producing cells (determined by IL17 Elispot assay) at baseline and the change in quantitative score for synovial thickening (10 MCP Trans STA score) from baseline to week 1 (**b**), from baseline to week 4 (**c**) and from baseline to week 12 (**d**) on treatment in the whole RA cohort. Correlation was tested using Spearman’s rank method. *spSFC/10* ^*6*^ specific spot forming cells per 10^6^, *10 MCP Trans PDA* composite transverse power Doppler area score for synovial vascularity of ten metacarpophalangeal joints

We investigated whether there was a relationship between higher frequencies of circulating Th17 cells prior to anti-TNF initiation and ultrasonographic measures of treatment response. A positive correlation was observed between the frequency of IL17-producing cells at baseline (by IL17 Elispot) and the change in synovial vascularity by PDUS from baseline to week 1 on treatment (*r* = 0.46, *p* = 0.02) (Fig. 4a). There were also strong positive correlations between the frequency of circulating IL17-producing cells at baseline and the change in synovial thickening from baseline to week 1 (*r* = 0.72, *p* = 0.0004), from baseline to week 4 (*r* = 0.51, *p* = 0.01) and from baseline to week 12 on treatment (*r* = 0.52, *p* = 0.01) (Fig. 4b-d). These relationships demonstrate that higher baseline frequencies of Th17 cells are associated with a smaller improvement, or worsening, in synovial vascularity and synovial thickening during anti-TNF therapy. Thus they suggest that higher baseline frequencies of Th17 cells may be associated with a poorer anti-TNF treatment response. Similarly, there was a positive correlation between the percentage of CD4 + IL17+ cells (by FACS) at baseline and the change in synovial vascularity score from baseline to week 1 (*r* = 0.66, *p* = 0.002) (Fig. 2, Additional file 2: Figure S2). There were also positive correlations between the percentages of CD4 + IL17+ cells at baseline and the change in synovial thickening score from baseline to week 1 (*r* = 0.51, *p* = 0.01), from baseline to week 4 (*r* = 0.36, *p* = 0.04) and from baseline to week 12 (*r* = 0.35, *p* = 0.05, Fig. 2, Additional file 2: Figure S2). The consistency of the results obtained using both Elispot and FACS adds strength to the findings.

In view of the relationships observed between a higher frequency of Th17 cells at baseline and a smaller improvement, or worsening, in synovial thickening and vascularity during treatment, we compared the frequency of circulating Th17 cells prior to anti-TNF initiation between EULAR good responders and non-responders to therapy. Anti-TNF non-responders showed a trend towards having a higher frequency of circulating Th17 cells at baseline compared to good responders, both by Elispot (EULAR non-responders vs good responders median (range) spSFC/10^6^, 538 (280–725) vs 405 (310–540), *p* value not significant) and FACS (EULAR non-responders vs good responders median (range) %, 0.9 (0.7–1.2) vs 0.7 (0.48–0.9), *p* value not significant).

## Discussion

We conducted a longitudinal investigation of patients with RA during the initial 12 weeks of anti-TNF treatment using clinical, ultrasonographic and T cell assessments to gain an understanding of immune correlates of treatment response. This longitudinal evaluation allowed us to identify a link between changes in circulating Th17 cells, evaluated by cellular assays, and resolving synovial inflammation and vascularity during anti-TNF treatment.

Anti-TNF treatment led to a significant and sustained improvement in clinical measures of disease activity and morphological improvement in synovial thickening and vascularity determined by grey scale and PDUS during 12 weeks of treatment. We observed strong positive correlations between DAS28, a composite measure of disease activity, and synovial vascularity score by PDUS, a more objective and quantitative measure of synovitis in the limited set of joints assessed. These findings are in agreement with previous studies [14–16, 30–33]. There was a clear difference between anti-TNF EULAR good responders and non-responders in the change in ultrasound measures of synovial thickening and vascularity during anti-TNF treatment. Responders demonstrated a significant improvement in synovial thickening and vascularity after 1, 4 and 12 weeks on treatment, whereas there were no significant changes in the non-responder group. The ultrasound measures of synovial vascularity were better able to discriminate between responder and non-responder groups compared to synovial thickening, which has also been shown by others [19, 29, 31, 34].

Synovial thickness and vascularity scores improved during anti-TNF treatment in EULAR good responders, but interestingly they exhibited different kinetics of change, with synovial vascularity showing earlier and more marked improvement compared with synovial thickening scores. PDUS signal has been shown to reflect vascularisation of pannus in RA and to correlate with histological changes of synovitis and synovial membrane microvascular density [32, 33]. One of the mechanisms of action of anti-TNF agents is through reduction of neovascularisation and angiogenesis of the synovial tissue by reducing expression of vascular endothelial growth factor (VEGF) [35]. Thus, anti-TNF appears to act rapidly to reduce synovial vascularity and therefore inflammation, which is reflected by improvement in ultrasound measures of vascularity. The reduction in synovial thickness assessed by grey scale ultrasonography is a slower process as it is likely to represent a decrease in swelling and inflammation of the synovium, which is likely a combination of reduction in infiltration of inflammatory cells in the joints, reduced expression of inflammatory cytokines and chemokines and reduction in synovial vascularity [36–38].

Using Elispot and intra-cellular cytokine staining, we demonstrated an increase in circulating Th17 cells during anti-TNF treatment in patients with RA. These results were obtained using two different but complementary techniques for assessing cellular immune responses and were consistent, thus strengthening our findings. The increase in circulating Th17 cells during anti-TNF treatment has been indicated in two small studies but these have evaluated Th17 cells using flow cytometry only, or by measurement of IL17 production by stimulated PBMC using ELISA at one time point on treatment [24, 25].

We found significant negative correlation between the change in numbers of Th17 cells from baseline to 12 weeks on treatment and the change in ultrasound scores for synovial thickening and vascularity from baseline to 12 weeks. Thus, as the frequency of Th17 cells increases in peripheral blood during anti-TNF treatment, there is a corresponding improvement in synovial thickening and vascularity. Our results suggest that the increase in Th17 cells in peripheral blood during treatment is associated with improvement in synovial thickening and vascularity.

This is the first study to link changes in T cell immunopathology assessed by cellular assays with the morphological changes in inflamed joints assessed by PDUS during anti-TNF treatment. These correlations are consistent with the mechanism of action of anti-TNF agents. One of the key mechanisms of action through which anti-TNF has been shown to lead to improvement in arthritis is through reduction in trafficking of inflammatory cells to joints through reduced synovial expression of chemokines and adhesion molecules and also reduced angiogenesis and synovial VEGF expression [35, 37, 38]. In patients with RA, infliximab causes a reduction in the cellularity of inflamed synovial tissue, with significant reductions in the number of intimal and sublining macrophages, plasma cells and T cells which parallels the rapid reduction in swollen joints as early as 48 hours after infliximab infusion [39]. Furthermore, a study in patients with RA demonstrated that the frequency of Th17 cells in synovial fluid from inflamed knee joints correlated with positive PDUS signal of the knee joint and increased levels of synovial fluid VEGF, suggesting that the presence of PDUS signal in the joints may therefore be a surrogate marker for the presence of Th17 cells [7]. Thus, the negative correlation between the increase in peripheral blood Th17 cells during anti-TNF treatment and the decrease in synovial thickness and vascularity on ultrasound suggests that anti-TNF treatment may induce redistribution of inflammatory cells from joints, leading to improvement in joint swelling and inflammation.

Another possible mechanism through which anti-TNF may cause an increase in circulating Th17 cells is through an increase in the p40 subunit shared between IL12 and IL23, the key cytokines involved in differentiation of Th1 and Th17 cells, respectively. In a study by our group using the CIA mouse model of RA, anti-TNF therapy ameliorated arthritis by decreasing numbers of Th1 and Th17 cells in arthritic joints, but also caused an increase in Th1 and Th17 cells in draining lymph nodes [23]. By using knockout mice, the increase in Th1 and Th17 cells was shown to occur through signalling via the TNFp55 receptor, which increased expression of the p40 subunit shared between IL12 and IL23. A similar mechanism was found to occur in a mouse model of reactive arthritis, where *Yersinia*-induced reactive arthritis in mice lacking TNFp55 receptor was associated with more severe disease. Increased levels of IL17, IL23 and IL12p70 were found in the arthritic joints of these mice and antibody blockade of IL17 was shown to reduce arthritis severity. The increase in Th17 responses in the TNFRp55-/- mice was shown to be mediated by an increase in IL12/23p40 [40]. TNFα-mediated inhibition of IL12/23p40 may also occur in human disease. A study by our group in patients with RA treated with anti-TNF showed that the increase in circulating Th17 cells up to 12 weeks on anti-TNF was accompanied by an increase in IL12/23p40 production in supernatants from PBMC stimulated with lipopolysaccharide (LPS) and also in the plasma layer of whole blood stimulated with LPS [25]. Taken together, these findings suggest that anti-TNF agents may act through several mechanisms to increase circulating Th17 cells during treatment.

Another interesting finding to emerge from this study is that anti-TNF non-responders showed a trend towards a higher baseline frequency of Th17 cells compared to responders and this trend was observed using results from both Elispot and intra-cellular cytokine staining. Two other studies also point to an association between higher baseline levels of IL17 or a higher frequency of Th17 cells and poor anti-TNF treatment response in RA; although in these studies this relationship has been investigated using clinical measures of disease activity only and at a single time point on treatment, rather than longitudinally [25, 41]. We investigated this hypothesis by exploring relationships between ultrasonographic and T cell immunological changes during anti-TNF therapy to determine if a higher frequency of IL17-producing cells at baseline was associated with poor treatment response. Indeed, we found significant correlation between higher numbers of Th17 cells at baseline and a smaller improvement in synovial vascularity on ultrasound at 1 week, and a smaller improvement in synovial thickening at 1, 4 and 12 weeks after anti-TNF initiation This suggests that a higher frequency of Th17 cells at baseline is associated with poor anti-TNF treatment response.

We have used two different but complementary techniques (Elispot and intracellular cytokine staining) to assess the frequency of Th17 cells prior to anti-TNF initiation and using both methods, we found a significant relationship between a higher baseline frequency of Th17 cells and poor treatment response assessed by ultrasonographic measures. If these associations are also confirmed in larger patient cohorts and in patients with other types of inflammatory arthritis aside from RA, the characterisation of Th17 cells as a marker of anti-TNF non-response raises the possibility of being able to tailor biologic therapy of inflammatory arthritis according to individual patient immunological profiles. Further investigation is warranted into whether patients with higher baseline frequencies of circulating Th17 cells may have more IL17-driven disease and whether these patients may derive greater benefit from treatment with a combination of anti-IL17 and anti-TNF agents.

## Conclusions

The present study shows that anti-TNF treatment induces an increase in circulating Th17 cells in patients with RA and this increase correlates with reduction in synovial thickening and vascularity. This is the first study to link increases in circulating Th17 cell numbers and IL17 production with resolution of ultrasonographic features of synovitis during anti-TNF treatment. We also showed that higher baseline frequencies of Th17 cells were associated with poorer anti-TNF treatment response. These observations raise the possibility that dual inhibition of IL17 and TNF may be a means to further advance therapeutic outcomes [42]. Fischer et al. have reported synergistic effects of TNF and IL-17 blockade in mice transgenic for human TNF and combined anti-TNF and IL-17 treatment had a superior effect on bone homeostasis than anti-TNF alone or anti-IL-17 alone [43].

## Additional files

Additional file 1: Figure S1. Depletion of CD4+ cells attenuates IL-17 responses in the Elispot assay. Cells were stained with CD4-PE pre and post magnetic labelling to assess effectiveness of CD4+ depletion by flow cytometry (**A** and **B**). Whole PBMCs prior to depletion contain CD4+ T cells (**A**) whilst after depletion of CD4+ T cells with magnetic beads, there are virtually no CD4+ T cells present in this representative patient (**B**). The frequency of IL17-producing peripheral blood mononuclear cells is markedly attenuated when CD4+ cells are depleted from the PBMC population (**C**). The effects of depletion are shown in three representative patients. PBMC (*n* = 200,000) prior to and after the depletion from each patient were set up in the IL17 Elispot assay and stimulated with 1 mg/ml anti-CD3 antibody (OKT3 clone). Results are expressed as number of IL17 specific spot forming cells per million PBMC. Representative experimental wells of the IL17 Elispot assay from one patient are shown demonstrating that after depletion of CD4+ cells the number of IL17-positive spots per well is attenuated (**D**). Each spot represents one cytokine-producing cell. The numbers under each well indicate the total number of IL17-positive spots in that well. *spSFC/10* ^*6*^ specific spot-forming cells per million PBMC. (DOCX 127 kb)
Additional file 2: Figure S2. Higher frequency of Th17 cells at baseline associated with poorer response to anti-TNF treatment. Positive correlations are shown between percentages of circulating CD4 + IL17+ cells (determined by intracellular cytokine staining using flow cytometry) at baseline and the change in quantitative score for synovial vascularity (10 MCP Trans PDA score) from baseline to week 1 on treatment (**A**) in patients with RA with presence of power Doppler signal at baseline (*n* = 19). Also positive correlations are shown between the percentages of circulating CD4 + IL17+ cells at baseline and the change in quantitative score for synovial thickening (10 MCP Trans STA score) from baseline to week 1 (**B**), from baseline to week 4 (**C**) and from baseline to week 12 (**D**) on treatment in the whole RA cohort. Correlations was tested using Spearman’s rank method. 10 MCP Trans PDA composite transverse power Doppler area score for synovial vascularity of ten metacarpophalangeal joints, 10 MCP Trans STA composite transverse synovial thickness area score of ten metacarpophalangeal joints. (DOCX 51 kb)

## Abbreviations

Anti-CCP: Anti-cyclic citrullinated peptide antibody
Anti-TNF: Anti-tumor necrosis factor
CIA: Collagen-induced arthritis
CRP: C-reactive protein
DAS28: Disease activity score of 28 joints
DMARDs: Disease-modifying anti-rheumatic drugs
ELISA: Enzyme-linked immunosorbent assay
ELISPOT: Enzyme-linked immunospot assay
ESR: Erythrocyte sedimentation rate
EULAR: European League Against Rheumatism
FACS: Fluorescence-activated cell sorting
IL17: interleukin 17
LPS: Lipopolysaccharide
MCP: Metacarpophalangeal joints
PBMC: Peripheral blood mononuclear cell
PDA: Power Doppler area
PDUS: Power Doppler ultrasound
RA: Rheumatoid arthritis
RPMI: Roswell Park Memorial Institute medium
spSFC: Specific spot forming cells
STA: Synovial thickness area
Th17: T helper 17 cells
VEGF: Vascular endothelial growth factor
