# Supplementary figures and images for: Increase in circulating Th17 cells during anti-TNF therapy is associated with ultrasonographic improvement of synovitis in rheumatoid arthritis
Source: Arthritis Res Ther. 2016 Dec 23;18:303. doi: 10.1186/s13075-016-1197-5 (PMC5180397; doi:10.1186/s13075-016-1197-5)

Additional file 1: Figure S1


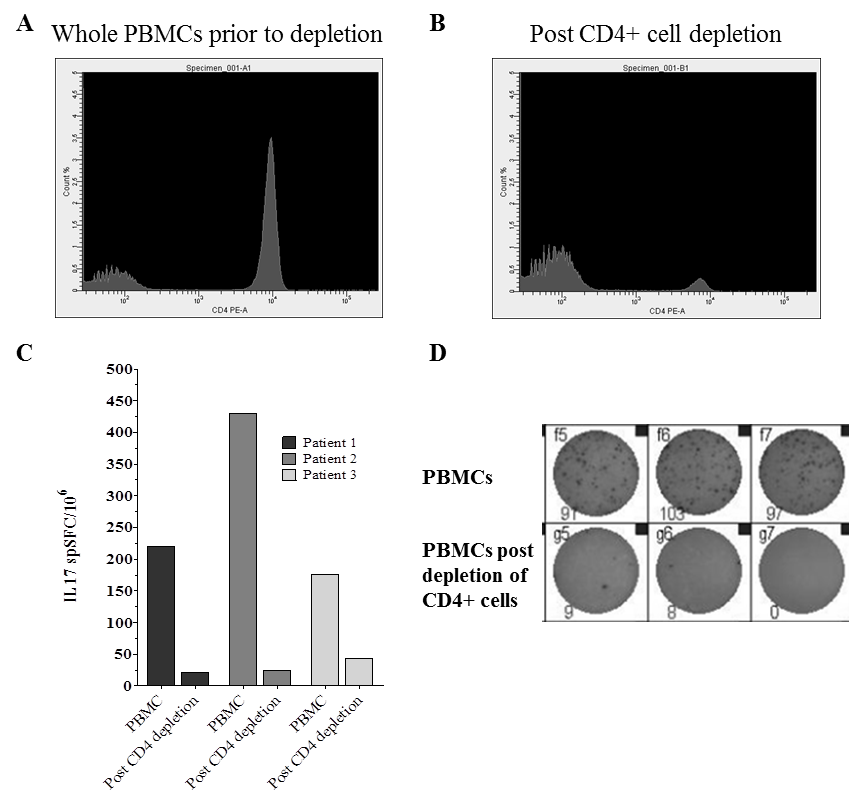

Supplement: Additional file 1: Figure S1. — Depletion of CD4+ cells attenuates IL-17 responses in the Elispot assay. Cells were stained with CD4-PE pre and post magnetic labelling to assess effectiveness of CD4+ depletion by flow cytometry (A and B). Whole PBMCs prior to depletion contain CD4+ T cells (A) whilst after depletion of CD4+ T cells with magnetic beads, there are virtually no CD4+ T cells present in this representative patient (B). The frequency of IL17-producing peripheral blood mononuclear cells is markedly attenuated when CD4+ cells are depleted from the PBMC population (C). The effects of depletion are shown in three representative patients. PBMC (n = 200,000) prior to and after the depletion from each patient were set up in the IL17 Elispot assay and stimulated with 1 mg/ml anti-CD3 antibody (OKT3 clone). Results are expressed as number of IL17 specific spot forming cells per million PBMC. Representative experimental wells of the IL17 Elispot assay from one patient are shown demonstrating that after depletion of CD4+ cells the number of IL17-positive spots per well is attenuated (D). Each spot represents one cytokine-producing cell. The numbers under each well indicate the total number of IL17-positive spots in that well. spSFC/10 6 specific spot-forming cells per million PBMC. (DOCX 127 kb) [file 13075_2016_1197_MOESM1_ESM.docx]

Additional file 2: Figure S2


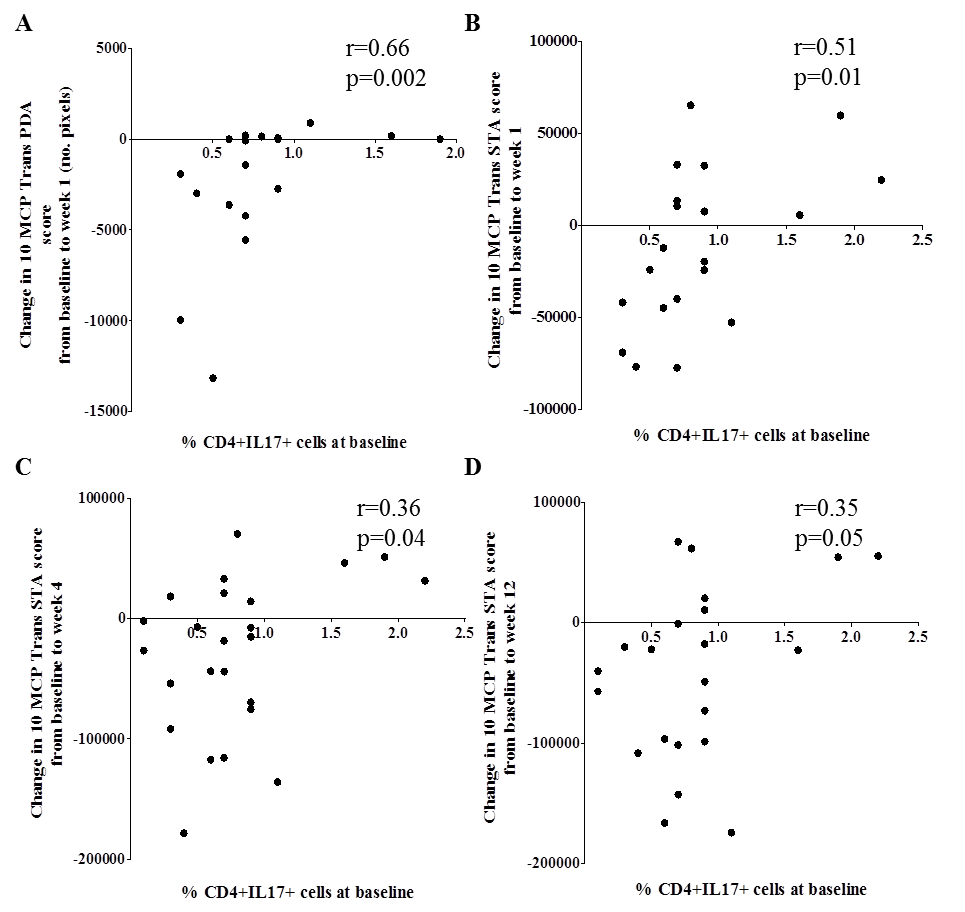

Supplement: Additional file 2: Figure S2. — Higher frequency of Th17 cells at baseline associated with poorer response to anti-TNF treatment. Positive correlations are shown between percentages of circulating CD4 + IL17+ cells (determined by intracellular cytokine staining using flow cytometry) at baseline and the change in quantitative score for synovial vascularity (10 MCP Trans PDA score) from baseline to week 1 on treatment (A) in patients with RA with presence of power Doppler signal at baseline (n = 19). Also positive correlations are shown between the percentages of circulating CD4 + IL17+ cells at baseline and the change in quantitative score for synovial thickening (10 MCP Trans STA score) from baseline to week 1 (B), from baseline to week 4 (C) and from baseline to week 12 (D) on treatment in the whole RA cohort. Correlations was tested using Spearman’s rank method. 10 MCP Trans PDA composite transverse power Doppler area score for synovial vascularity of ten metacarpophalangeal joints, 10 MCP Trans STA composite transverse synovial thickness area score of ten metacarpophalangeal joints. (DOCX 51 kb) [file 13075_2016_1197_MOESM2_ESM.docx]
